# Glutathione *S*-Transferase Polymorphisms, Passive Smoking, Obesity, and Heart Rate Variability in Nonsmokers

**DOI:** 10.1289/ehp.11402

**Published:** 2008-07-18

**Authors:** Nicole M. Probst-Hensch, Medea Imboden, Denise Felber Dietrich, Jean-Claude Barthélemy, Ursula Ackermann-Liebrich, Wolfgang Berger, Jean-Michel Gaspoz, Joel Schwartz

**Affiliations:** 1 Molecular Epidemiology/Cancer Registry, University of Zürich, Zürich, Switzerland; 2 Institute of Social and Preventive Medicine, University of Basel, Basel, Switzerland; 3 Laboratoire de Physiologie Clinique et de l’Excercie, Université Jean Monnet, Saint-Etienne, France; 4 Division of Medical Molecular Genetics and Gene Diagnostics, Institute of Medical Genetics, University of Zürich, Zürich, Switzerland; 5 Division of Primary Care Medicine, University Hospitals, Geneva, Switzerland; 6 Department of Environmental Health, Harvard School of Public Health, Boston, Massachusetts, USA

**Keywords:** cohort, glutathione *S*-transferase, heart rate variability, obesity, oxidative stress, polymorphism, SAPALDIA, second-hand smoke

## Abstract

**Background:**

Disturbances of heart rate variability (HRV) may represent one pathway by which second-hand smoke (SHS) and air pollutants affect cardiovascular morbidity and mortality. The mechanisms are poorly understood.

**Objectives:**

We investigated the hypothesis that oxidative stress alters cardiac autonomic control. We studied the association of polymorphisms in oxidant-scavenging glutathione *S*-transferase (*GST*) genes and their interactions with SHS and obesity with HRV.

**Methods:**

A total of 1,133 nonsmokers > 50 years of age from a population-based Swiss cohort underwent ambulatory 24-hr electrocardiogram monitoring and reported on lifestyle and medical history. We genotyped *GSTM1* and *GSTT1* gene deletions and a *GSTP1* (Ile105Val) single nucleotide polymorphism and analyzed genotype–HRV associations by multiple linear regressions.

**Results:**

Homozygous *GSTT1* null genotypes exhibited an average 10% decrease in total power (TP) and low-frequency-domain HRV parameters. All three polymorphisms modified the cross-sectional associations of HRV with SHS and obesity. Homozygous *GSTM1* null genotypes with > 2 hr/day of SHS exposure exhibited a 26% lower TP [95% confidence interval (CI), 11 to 39%], versus a reduction of −5% (95% CI, −22 to 17%) in subjects with the gene and the same SHS exposure compared with *GSTM1* carriers without SHS exposure. Similarly, obese *GSTM1* null genotypes had, on average, a 22% (95% CI, 12 to 31%) lower TP, whereas with the gene present obesity was associated with only a 3% decline (95% CI, −15% to 10%) compared with nonobese *GSTM1* carriers.

**Conclusions:**

GST deficiency is associated with significant HRV alterations in the general population. Its interaction with SHS and obesity in reducing HRV is consistent with an impact of oxidative stress on the autonomous nervous system.

Heart rate variability (HRV) is a noninvasive measure reflecting autonomic cardiac function that independently predicts death and arrhythmic complications in apparently healthy middle-age and elderly subjects (Task Force of the European Society of Cardiology and the North American Society of Pacing and Electrophysiology 1996; Tsuji et al. 1994, 1996). Disturbances in autonomous nervous system function reflected by decreased HRV may represent one pathway by which tobacco smoke, including second-hand smoke (SHS), and air pollutants trigger cardiovascular mortality and morbidity (Pope et al. 2001, 2004). The specific mechanisms by which these inhalants affect neural control of the heart are the focus of ongoing research. One potentially important pathway is oxidative stress (Brook et al. 2003; Cascio 2005; Nel 2005), because inhaled smoke (and other pollutants) provokes oxidative stress and an inflammatory response in the lung and heart (Donaldson et al. 2005; Gurgueira et al. 2002; Zhang et al. 2001, 2002). Although reactive oxygen species (ROS) have an established importance in the pathogenesis of cardiovascular diseases (Dhalla et al. 2000), their specific impact on autonomous nervous system activity and its reaction to inhalants remains to be established. Recent studies provide evidence for oxidative stress as one of the mechanisms for the effect of air pollution on HRV (Cascio 2005). We have demonstrated that the acute effect of particulate matter (PM) air pollution on HRV is modified by polymorphisms in the glutathione *S*-transferase (*GST*) gene *GSTM1* (Schwartz et al. 2005) and the hemochromatosis gene *HFE* (Park et al. 2006), both exhibiting antioxidative properties (Forsberg et al. 2001; Hayes and Strange 2000; Park et al. 2006). Common polymorphisms in *GST* genes were previously found to modify respiratory effects of inhaled toxicants in children, asthmatics, smokers, and the general population (Gilliland et al. 2002; He et al. 2002; Kabesch et al. 2004; Romieu et al. 2004) and to interact with tobacco smoke to increase the risk of coronary heart disease (Tamer et al. 2004). Romieu et al. (2005) demonstrated that dietary supplementation with plant-derived n-3 polyunsaturated fatty acids, known for their antioxidative properties, abrogated the acute association between fine PM and decreased HRV in a cohort of elderly Mexicans. However, these studies have dealt with only acute exposure scenarios and short-term changes in HRV, whereas most studies have linked baseline HRV to cardiovascular risk (Task Force of the European Society of Cardiology and the North American Society of Pacing and Electrophysiology 1996; Tsuji et al. 1994, 1996).

To further investigate the hypothesis of an ROS impact on autonomous nervous system activity in the general population, we have investigated the association between polymorphisms in *GSTM1*, *GSTT1*, and *GSTP1* and changes in HRV in male and female participants in the population-based SAPALDIA cohort (Swiss Cohort on Air Pollution and Lung and Heart Disease in Adults) ≥ 50 years of age. To indirectly test whether the effects of SHS were mediated by oxidative stress, we also assessed the interaction of SHS with *GST* polymorphisms and obesity.

## Materials and Methods

### Study population

SAPALDIA was designed to measure the health effects of air pollutants and has been previously described (Ackermann-Liebrich et al. 2005; Downs et al. 2007; Martin et al. 1997). Briefly, random samples of the Swiss population were recruited from eight areas featuring distinct geographic and environmental conditions. Participants were examined in 1991 and in 2001–2003. A random sample of follow-up participants ≥ 50 years of age participated in a 24-hr electrocardiogram recording (*n* = 1,837) (Felber Dietrich et al. 2006, 2007), including 1,133 nonsmoking subjects [see Supplemental Material (http://www.ehponline.org/members/2008/11402/suppl.pdf) for exclusion criteria]. The study protocol complied with all applicable ethical regulations. Participants gave written informed consent before the study. The study was approved by the Ethical Committee of the Swiss Academy of Medical Sciences and the eight cantonal ethical committees.

### Interview, body mass index, and blood pressure

SHS exposure was assessed at the in-person interview by asking subjects how many hours per day they were exposed to other people’s tobacco smoke (*a*) at home, (*b*) at the workplace, (*c*) in bars and restaurants, or (*d*) elsewhere. We focused on SHS exposure at home and work because these two sources dominate overall exposure in most subjects. We classified subjects as not exposed, exposed ≤ 2 hr/day, or exposed > 2 hr/day (Felber Dietrich et al. 2007). Weight, height, and body mass index (BMI; kg/m^2^) were based on measuring participants without them wearing any shoes or coats. Blood pressure was measured at rest in the sitting position on the left upper arm by an automatic device (705CP; OMRON, Steinhausen, Switzerland).

### HRV parameters

For 24-hr electrocardiogram (Holter) recording, digital devices (Aria, Del Mar Medical Systems, Irvine, CA, USA) with a frequency response of 0.05–40 Hz and a resolution of 128 samples/sec were used (Felber Dietrich et al. 2006, 2007). The recorders were hooked up after the interview. Participants were asked to follow their regular daily life and to fill in a time–activity diary during recording time. Mean duration of the recordings was 22.3 ± 2.1 hr. All recordings were scanned through a StrataScan 563 (Del Mar). Mean heart rate per minute was derived from Holter measurements. Spectral analysis was performed by the fast Fourier transform method. Here we focus on the frequency-domain variables because they allow resolution of total HRV [total power (TP); ≤ 0.40 Hz] into a component primarily reflecting parasympathetic stimulation [high-frequency (HF) power; 0.15–0.40 Hz] and a component reflecting both sympathetic and parasympathetic influences [low-frequency (LF) power; 0.04–0.15 Hz] [for methodological details, see Supplemental Material (online at http://www.ehponline.org/members/2008/11402/suppl.pdf); for results on time-domain parameters SDNN (standard deviation of all normal-to-normal intervals), SDANN (deviations of the normal-to-normal R-R period), and rMSSD (square root of the mean squared differences of successive R-R intervals), see Supplemental Material, Tables 1 and 2].

### Blood markers and genotype

All subjects were genotyped for *GSTM1* (UniGene ID Hs.301961; UniGene 2008a) and *GSTT1* (UniGene Hs.268573; UniGene 2008b) gene deletions and a single nucleotide polymorphism (SNP) in *GSTP1* (UniGene Hs.523836; UniGene 2008c) leading to the amino acid substitution Ile105Val, as previously described (Imboden et al. 2007) [for details, see Supplemental Material (online at http://www.ehponline.org/members/2008/11402/suppl.pdf)].

### Statistical analysis

We assessed the association of log-transformed HRV with *GST* genotypes, obesity, and SHS exposure by multiple regression analyses adjusting for study area, age (and its square), sex, diabetes, beta-blocker intake, and fruit intake. We present results as percent change in HRV parameters compared with the respective reference groups. To assess the interactions between SHS, *GST* genotypes, and obesity, we calculated trend tests by entering cross-categorized variables into the respective regression models. We coded the cross-categorized variables as 1 if both at-risk characteristics were absent, 2 if only one at-risk characteristic was present, and 4 if both at-risk characteristics were present in a subject. We performed statistical analysis using the software package SAS version 8.2 (SAS Institute, Inc., Cary, NC, USA) [for details, see Supplemental Material (http://www.ehponline.org/members/2008/11402/suppl.pdf)].

## Results

Table 1 presents characteristics of the study population, which have also been reported in more detail elsewhere (Felber Dietrich et al. 2007). In brief, 52% of the subjects included in the study were females. Mean age was 60.6 (SD 6.3) years, and mean BMI was 26.6 (4.2) kg/m^2^. Non-log-transformed means (SDs) for the different frequency-domain HRV parameters were TP, 4,583.1 msec^2^ (2,902.5); HF, 114.4 msec^2^ (235.0); and LF, 304.0 msec^2^ (275.9). SHS exposure either at work or at home was reported by 16% of the participants. In the present subpopulation of nonsmokers, 52% and 18% of subjects exhibited homozygous *GSTM1* and *GSTT1* gene deletion, respectively. Genotype distribution for the *GSTP1* Ile105Val SNP was 49% (Ile/Ile), 43% (Ile/Val), and 8% (Val/Val).

Table 2 presents the independent associations of *GST* genotypes, SHS, and obesity with changes in LF, HF, and TP HRV parameters. We observed no association for any of the predictors with HF values. *GSTT1* deficiency, > 2 hr/day of SHS exposure, and obesity were each independently associated with lower TP and LF, and there was a trend toward an association of *GSTP1* (Ile105Val) with TP. TP and LF were each 10% lower among subjects homozygous for *GSTT1* gene deletion compared with participants without the deletion (*p* = 0.02 and 0.04). The associations between HRV and *GSTP1* were consistent with the *GSTT1* findings but did not reach statistical significance. *GSTM1* deficiency was not associated with changes in any HRV parameter. SHS (−17.6%, *p* = 0.006) and obesity (−15.0%, *p* = 0.0003) were associated with larger reductions in overall HRV (TP) than were the genotypes. The associations of *GST* genotypes, obesity, and SHS with HRV time-domain parameters were generally consistent in direction with those observed for frequency-domain parameters, but reached statistical significance only for the obesity association with both SDNN and SDANN [see Supplemental Material, Table 1 (http://www.ehponline.org/members/2008/11402/suppl.pdf)].

**Figure 1:**
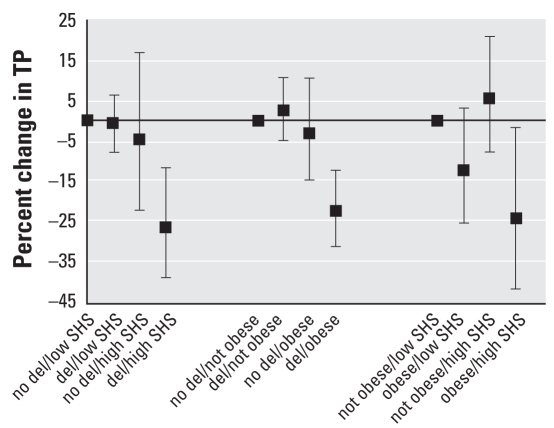
Combined effects (percent change and 95% CI) of *GSTM1* genotype [deletion (del) vs. no deletion], obesity (obese vs. not obese), and SHS (high SHS vs. no/low SHS) exposure on TP.

Table 3 and Figure 1 present the two-way interactive effects of *GST* genotypes, SHS, and obesity on HRV frequency-domain parameters. To maximize power to detect an interaction, for these analyses we characterized SHS exposure as either high (≥ 2 hr/day) or not. We found significant two-way interactions for the effect of high SHS exposure and *GSTM1*, *GSTT1*, *GSTP1*, and obesity on TP. For example, subjects with the *GSTM1* deletion and high SHS exposure had a 26.3% reduction in TP (95% CI, −38.7% to −11.6%) (Figure 1), and obese subjects with high SHS exposure had a 24.1% reduction in TP (95% CI, −41.5% to −1.5%) compared with *GSTM1* carriers and no or low SHS or absence of obesity, respectively (Figure 1). In addition, for LF, two-way interactions were also significant for *GSTT1*, *GSTP1*, and obesity. In contrast, we saw no interactions for HF.

The interaction between *GSTT1* and SHS was subadditive rather than superadditive, as shown in Table 3. Subjects with the gene and SHS had a 21.3% reduction in LF, subjects with the gene deletion but no/low SHS had a 12.1% reduction in TP, but subjects with both the high SHS exposure and the deletion only had a 6.7% reduction in TP.

Further, treating obesity as the exposure, we saw a significant two-way interaction with *GSTT1* for LF. In this case, the direction of the interaction was superadditive.

Although, generally, we observed no statistically significant interactions between *GST* genotypes, obesity, or SHS exposure and time-domain parameters, there was a suggestion for an elevated decrease in SDNN and SDANN among subjects exhibiting both *GSTM1* deletion genotype and high SHS exposure or obesity [see Supplemental Material, Table 2 (http://www.ehponline.org/members/2008/11402/suppl.pdf)].

The results presented above were unaltered when we adjusted them for any of the additional potential confounders listed under “Statistical analysis” (data not shown). Additional control for heart rate and systolic or diastolic blood pressure also did not materially alter these results. Additional analysis of HRV restricted to the sleep period according to diary information showed results similar to those achieved in the 24-hr analyses. On average, LF power was lower at night by 11.1% (*p* = 0.03) in the *GSTT1*-deficient group compared with the reference group.

## Discussion

We found associations between common *GST* gene variants that are involved in oxidant defense and HRV in the general population. Participants missing both copies of the *GSTT1* gene had, on average, 10% lower overall and LF-domain HRV. *GSTM1* deficiency and the *GSTP1* Ile105Val SNP were not independently associated with HRV changes, but we identified interactions between all three *GST* polymorphisms and exposure to SHS for effects on HRV. Combined with the interaction of SHS with obesity, a condition known to increase systemic oxidative stress, this provides support for the hypothesis that SHS affects HRV through oxidative stress pathways. This in turn implies that oxidative stress is an important modifier of the autonomic control of the heart, a hypothesis that has received little attention until recently.

The hypothesis of oxidative stress being a relevant pathophysiologic mechanism underlying individual variation in the functioning of the autonomous nervous system, and therefore HRV, is supported by (*a*) genetic polymorphisms related to oxidative defenses (Forsberg et al. 2001; Hayes and Strange 2000; Park et al. 2006) affecting HRV; (*b*) conditions likely to increase oxidative stress, such as obesity (Keaney et al. 2003) and SHS (Zhang et al. 2002), decreasing HRV; and (*c*) prooxidative conditions such as obesity and SHS interacting with genetic polymorphisms related to oxidative defenses.

Epidemiologic support for an association between oxidative stress and the autonomic control of the heart is still limited, but recent evidence is supportive of this hypothesis. Data in men from the Normative Aging Study recently provided strong evidence that oxidative stress may be a key pathway for the adverse effects of combustion particles on HRV (Schwartz et al. 2005). The association between fine PM and reduced HRV was restricted to persons missing the *GSTM1* gene and persons likely to have greater than average baseline systemic inflammation and oxidative stress, such as the obese. Statins, a widely prescribed class of lipid-lowering drugs with substantial antiinflammatory and antioxidant activity, protected *GSTM1*-deficient subjects against the effects of fine PM (Schwartz et al. 2005). One key difference in our study is that we aimed to assess the effect of a chronic exposure (SHS, obesity) on baseline HRV, rather than an acute one. Although some of the observed associations can still be attributed to acute effects due to collinearity between chronic and acute exposure, the association with chronic exposure suggests an ongoing and not a transitory impact on HRV, which may have more public health relevance. On the other hand, the similarity between SHS and ambient PM is sufficiently high that the finding of interactions of *GSTM1* status and obesity with these exposures in two separate cohorts argues against this being a chance finding.

The association of chronic stimuli such as obesity, insulin resistance, and diabetes with reduced HRV (Furukawa et al. 2004) is also compatible with an ROS impact on the autonomic nervous system. In nondiabetic human subjects, fat accumulation and obesity are closely correlated with markers of systemic oxidative stress (Keaney et al. 2003; Olusi 2002). Diabetic patients are known to have elevated oxidative stress levels, and they also exhibit increased susceptibility to the effect of air pollution on HRV (Zanobetti and Schwartz 2002). Chronic administration of the antioxidant vitamin E in a double-blind randomized controlled trial in patients with type 2 diabetes and cardiac autonomic neuropathy improved the ratio of the cardiac sympathetic to parasympathetic tone (Manzella et al. 2001). Marine- and plant-derived omega-3 fatty acid supplementation in elderly nursing home residents was associated with a significant increase in HRV (Holguin et al. 2005). The omega-3 fatty acid effects are possibly attributable in part to their antioxidative properties (Mori et al. 2003).

The exact mechanisms by which ROS and associated inflammatory mediators affect the autonomous nervous system and its correlate HRV are still poorly understood and likely complex. Oxidants and inflammatory mediators can act directly in the brain, as evidenced by the involvement of oxidative stress in various neurodegenerative diseases such as Alzheimer’s and Parkinson’s disease (Chong et al. 2005). Inflammatory markers, including interleukin-6, are present in the brain, where they can influence the autonomic balance (Juttler et al. 2002). A central nervous system effect of ROS is also compatible with results from a recent investigation in spontaneously hypertensive rats that are characterized by elevated oxidative stress (Girouard et al. 2004). The antioxidants *N*-acetylcysteine and melatonin restored cardiac baroreflex to normal, but not blood pressure, an effect that could be attributable to a central nervous system ROS effect. Interestingly, these rats were characterized by a primarily sympathetic defect, and our study finds the effects of SHS, obesity, and *GST* polymorphisms are absent for HF, which reflects a primarily parasympathetic response.

Finally, ROS and inflammatory markers may further exacerbate the autonomic disturbances on the heart through peripheral local effects on heart structures (Lee and Widdicombe 2001), because oxidative stress is the most commonly hypothesized mechanism by which several cytotoxic anticancer drugs cause cardiotoxicity (Stone and Godleski 1999). Tracey (2002) describes the central role of the autonomous nervous system in monitoring as well as regulating oxidative stress and inflammation at innervated pulmonary and extrapulmonary sites as the inflammatory reflex. In accordance with the inflammatory reflex model and our results, recent studies in rats intratracheally exposed to urban PM suggested a pulmonary-to-cardiac signaling model with pulmonary oxidants increasing cardiac oxidant concentrations under the strict control of the autonomous nervous system. Cardiac oxidative stress was preventable by both *N*-acetylcysteine and β_1_ receptor antagonist pretreatment of these animals (Rhoden et al. 2005).

The respective impact of the different *GST* polymorphisms on HRV observed in this study further elucidates ROS mechanisms. The *GST* genes and isozymes exhibit differences in tissue expression as well as substrate specificity (Forsberg et al. 2001; Hayes and Strange 2000). Although liver is the only rich source of the *GSTM1* isozyme, where it is the predominant form, *GSTP1* and *GSTT1* are expressed in various tissues, including heart, brain, lung, and liver (Hayes and Strange 2000; Rowe et al. 1997). The presence of *GSTT1* and—statistically nonsignificant—*GSTP1* main effects and the absence of a *GSTM1* main effect on HRV are consistent with an impact of locally and endogenously produced ROS in lung, heart, brain, and possibly additional organs on the activity of the autonomous nervous system. The hypothesis that endogenously produced oxidative stress affects the nervous system is further supported by the observation that lack of *GSTT1*, but not of other *GST* variants, is generally associated with increased susceptibility to brain diseases, including brain tumors and neurodegenerative diseases, even in apparently unexposed individuals (Landi 2000).

The modifying effect of *GSTM1* for the association of SHS with HRV suggests that these exposures cause systemic oxidative stress that is being scavenged by *GSTM1* in the liver. The modification of the SHS effect by *GSTM1* is consistent with previous studies on the association between SHS, *GST* polymorphisms, and lung cancer in never smokers (Wenzlaff et al. 2005) and may reflect the additional impact of *GSTM1* on liver metabolism of tobacco-derived electrophils (Hayes and Strange 2000; Landi 2000; Rowe et al. 1997). The modifying effect of *GSTP1* variants suggests that oxidative stress in target tissues other than liver is also important for the effects of SHS. The observation of subadditive effects of *GSTT1* and SHS, in contrast to the superadditive effects of *GSTT1* and obesity, suggest that some specific components of SHS drive the direction of the interaction. Depending on the substrate, *GSTT1*-catalyzed reactions can actually increase toxicity (Landi 2000). What that component is remains to be determined.

This study has a number of limitations. In all genetic studies, the prevalence of the polymorphism can limit power. A greater concern in this study is the 6.9% prevalence rate for high SHS exposure, which clearly limits power in gene-by-environment interactions. An additional limitation of this study is its cross-sectional design. We recorded electrocardiograms once for each subject. The future longitudinal assessment at the next follow-up examination will allow for improved adjustment of within-subject variation and allow us to examine differences in baseline autonomic function over time. The aging of the cohort will provide information on the combined impact of modifiable and genetic factors on the course of HRV decline and on the incidence of cardiac diseases. Finally, although the reported associations were comparable for frequency- and time-domain parameters, they were generally stronger and more consistent in the frequency domain.

However, the focus on frequency-domain parameters seems justified. First, the frequency-domain parameter HF captures the vagal, parasympathetic response more clearly than does rMSSD. Second, the Fourier transformation for TP, but not SDNN, is for a specified frequency range that trims off some ultra-HF signals. Extending the upper limit of the HF component, which is implicitly lacking in SDNN, beyond 0.4 Hz would be applicable only to extreme tachypnea of > 24 respiratory cycles per minute. This is linked to extreme sympathetic overdrive, under which it is rather difficult to interpret the HF component. Moreover, because the cardiac period signal is discrete rather than continuous, it is difficult to properly estimate respiratory arrhythmia under such conditions of very fast tachypnea.

In conclusion, our results are consistent with an important role of oxidative stress in the autonomic control of the heart and, possibly, in individual variability in autonomous nervous system activity. If confirmed by additional studies specifically investigating the association between systemic oxidative stress markers and HRV, these findings have substantial public health relevance. Tsuji et al. (1996) suggested that a 1-SD reduction in overall HRV was associated with a relative risk of 1.47 for cardiac events over 3.5 years of follow-up. Although differences in study design preclude a quantitative risk estimate, the observed reduction in overall HRV in subjects with SHS exposure (>2 hr) and either obesity, *GSTM1* deletion, or *GSTP1* substitution in our study suggests a nontrivial elevation of cardiovascular risk on follow-up, and one similar to what has in fact been reported for SHS exposure (Law and Wald 2003).

## Figures and Tables

**Table 1:** Characteristics of the study population: the SAPALDIA cohort study.

Characteristic	Value
Total [no. (%)]	1,133 (100.0)
Female sex [no. (%)]	594 (52.4)
Age [years (mean ± SD)]	60.6 ± 6.3
BMI [kg/m^2^ (mean ± SD)]	26.6 ± 4.2
≥ 30 kg/m^2^ [no. (%)]	223 (19.7)
SHS exposure [no. (%)]	
None	956 (84.5)
≤ 2 hr/day	99 (8.7)
> 2 hr/day	78 (6.9)
Diabetes [no. (%)]	41 (3.6)
Medication [no. (%)]	
Beta-blocker	135 (11.9)
ACE inhibitor	70 (6.2)
Antiarrhythmics, classes I + III	5 (0.4)
Calcium-channel blocker	53 (4.7)
Diuretics	42 (3.7)
Sympathomimetics	37 (3.3)
Uric acid [μmol/L (mean ± SD)]	323.6 ± 81.6
High-sensitivity C-reactive protein [mg/L (mean ± SD)]	2.5 ± 5.8
Non-HDL cholesterol [mmol/L (mean ± SD)]	4.7 ± 1.1
Blood pressure [mmHg (mean ± SD)]	
Systolic	132.5 ± 19.3
Diastolic	81.9 ± 10.6
Heart rate [bpm (mean ± SD)]	73.5 ± 9.0
HRV (mean ± SD)	
TP (msec^2^)	4583.1 ± 2902.5
LF power (msec^2^)	304.0 ± 275.9
HF power (msec^2^)	114.4 ± 235.0
SDNN (msec)	138.7 ± 36.5
SDANN (msec)	125.6 ± 35.0
rMSSD (msec)	26.2 ± 14.9
Genotypes [no. (%)]	
*GSTM1* deletion	594 (52.4)
*GSTT1* deletion	199 (17.6)
*GSTP1*	
Ile/Ile	550 (48.5)
Ile/Val	485 (42.8)
Val/Val	98 (8.7)

**Table 2:** Percent difference (95% CI)^a^ in HRV parameters according to *GST* genotypes, SHS, and obesity: the SAPALDIA cohort study.

Genotype/exposure	LF power	HF power	TP
*GSTM1*			
Deletion vs. no deletion^b^	−1.7 (−9.3 to 6.4)	−1.6 (−11.2 to 9.0)	−2.6 (−9.1 to 4.4)
*GSTT1*			
Deletion vs. no deletion^b^	−10.7 (−19.6 to −0.7)	−3.4 (−15.6 to 10.5)	−10.4 (−18.2 to −1.9)
*GSTP1* to Ile105Val			
Ile/Ile,Val vs. Val/Val	−7.9 (−20.1 to 6.2)	−8.6 (−23.8 to 9.7)	−10.6 (−20.9 to 1.1)
SHS exposure			
≤ 2 hr/day vs. none	−10.3 (−22.3 to 3.5)	−13.0 (−27.5 to 4.6)	−4.3 (−15.5 to 8.2)
> 2 hr/day vs. none	−16.4 (−28.8 to −1.9)	−2.6 (−20.6 to 19.6)	−17.6 (−28.3 to −5.4)
Obesity			
≥ 30 vs. < 30 kg/m^2^	−19.5 (−27.4 to −10.8)	−5.0 (−16.7 to 8.4)	−15.0 (−22.2 to −7.2)

a Adjusted for study area, sex, age and (age)^2^, fruit intake, diabetes, and beta-blocker intake and mutually adjusted for each other.

b Homozygous gene deletion.

**Table 3:** Percent difference (95% CI)^a^ in HRV parameters according to combination of *GST* genotypes with passive smoking and obesity: the SAPALDIA cohort study.

Genotype	Exposure	No.	LF power	HF power	TP
*GSTM1*					
No deletion	No/low SHS	504	Referent	Referent	Referent
No deletion	High SHS	35	−11.5 (−30.0 to 27.7)	−2.2 (−12.0 to 8.7)	−4.6 (−22.0 to 16.7)
Deletion^b^	No/low SHS	551	−1.2 (−9.0 to 7.3)	−5.5 (−30.0 to 27.7)	−0.8 (−7.7 to 6.4)
Deletion^b^	High SHS	35	−19.9 (−35.4 to −0.7)	1.6 (−22.8 to 33.9)	−26.3 (−38.7 to −11.4)
*p*-Value for trend^c^			0.094	0.86	0.014
No deletion	Not obese	437	Referent	Referent	Referent
No deletion	Obese	102	−13.3 (−25.3 to 0.7)	1.6 (−16.2 to 23.0)	−3.0 (−14.7 to 10.4)
Deletion^b^	Not obese	473	1.2 (−7.5 to 10.6)	1.0 (−16.2 to 23.0)	2.4 (−5.2 to 10.6)
Deletion^b^	Obese	121	−23.5 (−33.4 to −12.0)	−9.4 (−24.2 to 8.2)	−22.1 (−30.9 to −12.1)
*p*-Value for trend^c^			0.16	0.69	0.25
*GSTT1*					
No deletion	No/low SHS	871	Referent	Referent	Referent
No deletion	High SHS	63	−17.6 (−30.9 to −1.7)	−3.0 (−22.6 to 21.5)	−21.3 (−32.3 to −8.5)
Deletion^b^	No/low SHS	184	−11.1 (−20.2 to −0.7)	−4.1 (−16.7 to 10.3)	−12.1 (−20.0 to −3.5)
Deletion^b^	High SHS	15	−16.5 (−41.1 to 18.5)	6.9 (−31.8 to 67.4)	−6.7 (−30.9 to 25.9)
*p*-Value for trend^c^			0.007	0.77	0.002
No deletion	Not obese	748	Referent	Referent	Referent
No deletion	Obese	186	−18.1 (−26.7 to −8.4)	−5.3 (−18.0 to 9.3)	−15.1 (−22.9 to −6.4)
Deletion^b^	Not obese	162	−8.7 (−18.8 to 2.6)	−3.5 (−16.9 to 12.1)	−10.3 (−19.0 to −0.8)
Deletion^b^	Obese	37	−33.1 (−46.7 to −16.0)	−7.3 (−30.8 to 24.0)	−24.5 (−37.9 to −8.1)
*p*-Value for trend^c^			0.011	0.79	0.084
*GSTP1*, Ile105Val					
Val/Val	No/low SHS	87	Referent	Referent	Referent
Val/Val	High SHS	11	−16.9 (−45.9 to 27.7)	13.4 (−34.6 to 99.5)	−9.4 (−37.3 to 32.0)
Ile/Ile, Val	No/low SHS	969	−7.9 (−20.7 to 7.0)	−6.5 (−22.9 to 13.3)	−9.7 (−20.6 to 2.7)
Ile/Ile, Val	High SHS	67	−22.6 (−37.8 to −3.6)	−9.4 (−31.6 to 19.9)	−26.4 (−39.1 to −11.2)
*p*-Value for trend^c^			0.020	0.53	0.011
No deletion	Not obese	79	Referent	Referent	Referent
No deletion	Obese	19	−2.2 (−30.7 to 38.0)	6.6 (−31.4 to 65.6)	10.5 (−17.9 to 48.8)
Deletion^b^	Not obese	831	−4.1 (−18.1 to 12.4)	−6.3 (−23.5 to 14.7)	−5.5 (−17.5 to 8.4)
Deletion^b^	Obese	204	−24.1 (−36.5 to −9.2)	−11.9 (−29.9 to 10.8)	−21.8 (−32.8 to −8.5)
*p*-Value for trend^c^			0.073	0.66	0.33
Obesity					
No/low SHS	Not obese	855	Referent	Referent	Referent
No/low SHS	Obese	200	−1.2 (−18.0 to 19.1)	9.9 (−13.5 to 29.1)	−12.1 (−25.2 to 3.2)
High SHS	Not obese	55	−3.4 (−17.4 to 13.0)	5.6 (−13.7 to 29.1)	5.4 (−7.9 to 20.7)
High SHS	Obese	23	−44.4 (−58.9 to −24.8)	−18.6 (−44.9 to 20.0)	−24.1 (−41.5 to −1.5)
*p*-Value for trend^c^			0.002	0.76	0.049

a Adjusted for study area, sex, age and (age)^2^, fruit intake, diabetes, and beta-blocker intake and mutually adjusted for each other. We did not mutually adjust *GST* polymorphisms for each other. We adjusted *GST*/SHS models for BMI as a continuous variable, and adjusted *GST*/obesity models for SHS.

b Homozygous deletion.

c We derived the *p*-values for trend by entering a cross-categorized variable coded as 1, 2, and 4 for subjects exhibiting 0, 1, or 2 at-risk characteristics, respectively.
